# Relationship between body composition indices and changes in body temperature due to hot pack use

**DOI:** 10.1186/s40101-022-00313-0

**Published:** 2022-11-11

**Authors:** Kazuyuki Kominami, Kazunari Sato, Naoaki Takahashi

**Affiliations:** 1 Department of Rehabilitation, Sanseikai Kitano Hospital, 6-30, 1-Chome, Kiyota1-Jyo, Kiyota-Ku, Sapporo, 004-0861 Japan; 2 Health Sciences University of Hokkaido, Graduate School of Rehabilitation Sciences, 1757 Kanazawa, Tobetsu, Hokkaido 061-0293 Japan; 3 Health Sciences University of Hokkaido, Rehabilitation Sciences, 1757 Kanazawa, Tobetsu, Hokkaido 061-0293 Japan

**Keywords:** Thermal therapy, Hot pack, Body composition, Skin temperature, Deep-body temperature

## Abstract

**Background:**

Hot pack application is used to reduce pain and muscle stiffness at the treated site. However, the effects of hot pack application on the whole body have not been clarified. We investigated the relationship between body composition indices and the hot pack-induced increase in body temperature.

**Methods:**

We recruited 17 healthy men (age, 22.0 ± 3.3 years) who participated in the study on five different days and applied “dry” hot packs at four different sites (the most frequently used sites): right shoulder, lower back, both popliteal areas, and lower back plus popliteal areas. The study protocol involved the measurement of body composition followed by 10 min of bed rest, 15 min of warming with a hot pack, and 20 min of subsequent rest. Heart rate and body temperature were measured continuously, and blood pressure was recorded at 5-min intervals. Body temperature was measured at the right upper arm, precordium, abdomen, lumbus, right hallux, right femur, and right auditory canal.

**Results:**

Skin temperature increased significantly at and near the hot pack application site, but this finding showed no relationship with body composition indices. The warmability distal to the application site was negatively correlated with the body water content index. The auditory canal temperature did not change in any of the sessions.

**Conclusions:**

Hot pack usage alone did not increase the deep-body temperature and only increased the temperature around the application area. Moreover, higher body water content may allow for easier dissipation of heat from the peripheral extremities.

**Supplementary Information:**

The online version contains supplementary material available at 10.1186/s40101-022-00313-0.

## Background

Hot pack application is a form of thermal therapy that is widely and commonly applied for both medical and home use. In medical practice, hot packs are used in physical therapy for pain reduction and muscle relaxation [1–5]. In the nursing field, they are also used for pain management and relaxation [6–12]. The existing hot packs include cotton canvas bags containing water-soluble polymers that are heated in a thermostatic bath and wrapped with a towel, ceramic sheets or graphene heated by electricity, and sealed packages of silicone gel heated in a microwave oven.

The application of a hot pack heated to 80–85 °C in a thermostatic bath can be performed with “dry” and “wet” methods. In the “dry” method, the warmed hot pack is wrapped in a plastic bag and then double-wrapped in a bath towel. In the “wet” method, the heated hot pack is wrapped directly in three or four bath towels. Heat transfer in hot packs is generally known to occur through thermal conduction [1–3]. For this reason, hot pack application is classified as a superficial thermal therapy. The skin temperature just below the site of application of a hot pack heated to 80 °C in the “dry” method increases to nearly 40 °C after approximately 15 min of application [13, 14]. In addition, subcutaneous temperature increases by approximately +3 °C at 1 cm subcutaneously and +0.5 °C at 3 cm subcutaneously [3, 15, 16]. The use of a hot pack has been shown to decrease muscle stiffness and improve muscle reaction time in the applied muscle, in addition to improving joint range of motion and reducing pain. It also increases the blood flow in the applied area [17, 18].

Previous reports have shown an association between body fat mass and changes in the body temperature in hot and cold water baths [19]. However, only a few studies have examined the effects of hot pack use on parts of the body other than the application site, and the detailed effects in this regard have not been elucidated [20, 21]. Moreover, while the results showed an association between the increase in body temperature due to hot pack use and the amount of body fat, that is, between fat and heat conduction [20], the participants in those studies were mostly excessively obese individuals, thus providing little clinically meaningful information.

In recent years, a method that can measure body composition with a simple and detailed procedure by using multiple broadband frequencies (bioelectrical impedance analysis method: BIA method) has become popular. The BIA method allows for components of the human body (body water, protein, minerals, and body fat) to be quantitatively measured and analyzed from the impedance generated when an electric current is applied to the human body [22, 23]. In this study, we aimed to determine the changes in skin temperature and auditory canal temperature at the application site and other sites during the use of a dry hot pack and to determine whether body composition indices were related to the increase in body temperature. We hypothesized that the body composition indices obtained using the BIA method in healthy men of general body size would be related to the increase in body temperature due to hot pack use.

## Methods

In this observational study, we assessed the relationship between changes in body temperature and body composition indices during hot pack use and subsequent rest at five different sites. Seventeen healthy men over the age of 20 years (mean age, 22.0 ± 3.3 years; mean height, 172.4 ± 6.0 cm; mean weight, 65.5 ± 8.5 kg; mean body mass index [BMI], 22.0 ± 1.8 kg/m^2^) were recruited. The participants had no underlying medical conditions such as cardiac or respiratory diseases and were not receiving medical treatment for hypertension, diabetes mellitus, or dermatosis.

### Determination of sample size

We used G*power version 3 to calculate the sample size. The sample size calculated (analysis of variance [ANOVA]; effect size f, 0.25; *α* err prob, 0.05; power [1−*β* err prob], 0.8; number of measurements, 10) was 14. Therefore, we determined that the estimated sample size was 15. Finally, we enrolled 17 participants.

### Ethical considerations

The study was approved by the Ethics Committee of the Health Sciences University of Hokkaido (approval number: 19R100101). Informed consent was obtained from all participants for participation in the study and for the publication of this report.

### Hot pack thermal therapy

The participants were instructed to refrain from eating and smoking for 3 h before the thermotherapy session and from caffeine intake for 6 h before the session. All thermal therapy sessions were conducted in a quiet, private room, and the room temperature was adjusted to a constant 26–28 °C during the sessions. The participants wore T-shirts and knee-length shorts.

**Fig. 1:**
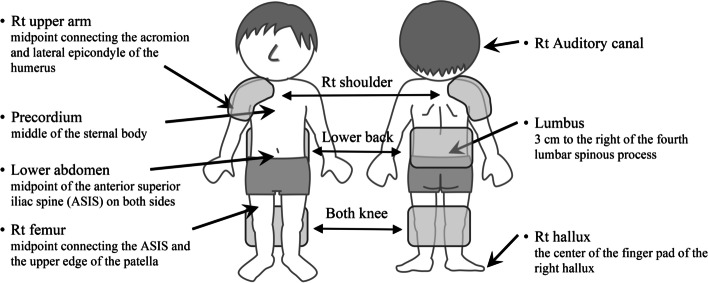
Body temperature measurement sites and hot pack thermal therapy application sites. The shoulder was wrapped so that the right acromion was in the center of the hot pack. The lumbar back was wrapped so that the fourth lumbar vertebra was at the center of the hot pack. For both knee fossae, both knees were wrapped so that the knee fossae became the center of the hot pack

Hot packs (Minato Pack, Large KPA1065 270×500mm; Minato Medical Science Co., Ltd., Osaka) were heated to 80 °C in a thermostatic bath (wet hot pack device, Pack Warmer CL-25; Sakai Medical Co., Ltd., Tokyo), wrapped in a plastic bag, and then wrapped in a bath towel for dry heat. The packs were applied to the right shoulder, lower back, both knee fossae, and a combination of the lower back and both knee fossae. For the right shoulder, the shoulder was wrapped so that the acromion was in the center of the hot pack. The lower back was wrapped so that the fourth lumbar vertebra was at the center of the hot pack. For both knee fossae, both knees were wrapped so that the knee fossae became the center of the hot pack (Fig. 1).

The order in which the hot packs were applied was determined using a random number table at the time of study enrollment. The interval between experiments was at least 24 h, and the second and subsequent experiments were conducted as soon as possible. All sessions were conducted within a maximum of 2 months.

### Study protocol

All sessions were conducted between 16:00 and 19:00. At each session, the participants underwent an evaluation of their physical condition followed by body composition measurements. Subsequently, the participants were placed in a supine position on a bed; body temperature sensors (details are provided in the next section) were attached to each part of the body; and a hot pack was applied to the target site. After 15 min of heating, the hot pack was removed, and the patient rested for another 20 min. The total protocol lasted for approximately 45 min. The time allocations were set based on general usage [1, 2] and our own previous research [24]. During the experiment, the skin temperature of each body part and auditory canal temperature as well as the heart rate were measured every second, and blood pressure was measured at 5-min intervals.

### Body temperature measurement

Body temperature measurements were performed at seven sites: the right auditory canal, the middle of the right upper arm (midpoint connecting the acromion and lateral epicondyle of the humerus), the precordium (middle of the sternal body), the lumbus (3 cm to the right of the fourth lumbar spinous process), lower abdomen (midpoint of the anterior superior iliac spine [ASIS] on both sides), right femur (midpoint connecting the ASIS and the upper edge of the patella), and the center of the finger pad of the right hallux (Fig. 1).

The auditory canal temperature, which approximates the tympanic temperature [25], was measured by inserting a tympanic temperature sensor (earplug type LT-2N-13; Gram Corporation, Saitama) into the right auricle. Skin temperature was measured by attaching a skin temperature sensor (LT-2N-12; Gram Co., Ltd., Saitama) to the skin at the measurement site with tape. The temperature change caused by the hot pack was also measured using an air temperature sensor (LT-2N-00; Gram Corporation, Saitama). A high-performance thermometer LT-200SA (Gram Corporation, Saitama) was used to record the measurement data.

### Heart rate and blood pressure

Heart rate was measured continuously throughout the session by using electrocardiography, and blood pressure (systolic, diastolic, and mean blood pressure) was measured at 5-min intervals using the oscillometric method. The locations of the electrocardiogram electrodes were based on CM5 [26, 27] and were adjusted to record the R wave clearly. R wave interval data were stored at a sampling rate of 1000 Hz using an LRR 03 (Crosswell, Yokohama, Japan) during both thermal therapies, and RR intervals were automatically calculated using Reflex Meijin (Crosswell, Yokohama, Japan).

### Body composition measurement

Body composition was measured before the administration of thermal therapy by using the body composition analyzer Inbody S10 (Inbody Japan Inc., Tokyo, Japan), which uses the BIA method. The BIA method is a technique that quantitatively measures the components of the human body from the impedance generated when an electric current is applied to the human body [22, 23]. The body composition indices measured in this study were extracellular water (ECW), intracellular water (ICW), total body water (TBW), ECW/TBW, protein mass (PM), soft lean mass (SLM), skeletal muscle mass (SMM), percent body fat (PBF), and the visceral fat area (VFA). Indicators related to body water content, such as ECW, ICW, and TBW, were also measured in five separate locations: both upper extremities, trunk, and both lower extremities.

### Estimation of the heat content of the hot pack

The dry and wet weights of the hot packs were measured, and the heat content was estimated from the components contained in the hot pack, the increased water content (specific heat: 4180 J/kg K), and the surface temperature of the hot pack. The hot packs had canvas (100 g, specific heat: 1,300 J/kg K) for the packing bag and bentonite (1140 g, specific heat 1700 J/kg K) [28] for the encasing material. In addition, the amount of heat emitted by the hot pack at a given time was calculated from the temperature decrease due to hot pack use.

### Statistical analysis

Data are presented as mean ± standard deviation. Statistical analyses were performed using Statistics for Excel 2012 (Social Survey Research Information Co., Tokyo). Body temperature and heart rate data were obtained at 5-min intervals starting from the end of baseline, through 5, 10, and 15 min of heating, followed by 5, 10, 15, and 20 min of rest. For each of these measurements, the average value of the immediately preceding 10 s was used as the data for each period. Changes in body temperature were tested using a one-way ANOVA using the end of baseline as the reference, and the Williams test was used as a post hoc test.

We also calculated the degree of increase in body temperature from the end of the baseline to the end of heating and the amount of decrease in body temperature from the end of heating to the end of rest. The correlations between the body composition indices obtained by the Inbody S10 and these indices were then established using Pearson’s correlation coefficient. For all statistical analysis, significance was defined as *p*<.05.

## Results

No adverse events, such as low-temperature injury, tympanic injury, and postural hypotension, were observed in this study. The body composition indices for each heating site are presented in Table 1. No significant differences in body composition indices or heart rate and blood pressure were observed in any of the sessions. The body temperature measurements showed no increase in auditory canal temperature, which reflects the deep-body temperature, regardless of the area to which the hot pack was applied. The skin temperature at and near the application site increased, but the skin temperature distal to the application site or on the contralateral side of the body (i.e., the ventral side when the application site was on the dorsal side) did not increase.

**Table 1:** Body composition data

		Total	Rt shoulder	Lower back	Lower back and both knees	Both knees	Control	*p* value
Age	[years]	21.9±3.1						
Height	[cm]	172.1±5.7						
Body weight	[kg]	65.1±8.0	64.4±8.0	64.4±8.3	64.7±7.8	64.9±7.8	65.1±8.0	0.999
BMI		21.9±1.7	21.7±1.8	21.7±1.9	21.7±1.8	21.7±1.9	21.9±1.8	0.997
ICW	[%]	25.9±3.5	25.8±3.4	25.6±3.5	25.9±3.5	26.0±3.7	26.0±3.9	0.413
ECW	[%]	15.1±2.0	15.0±1.9	15.0±2.0	15.1±2.0	15.2±2.1	15.1±2.1	0.933
TBW	[%]	41.0±5.5	40.9±5.3	40.7±5.5	41.0±5.5	41.2±5.7	41.1±6.0	0.653
ECW/TBW (Total)		36.9±0.6	37.0±0.4	37.1±0.4	37.1±0.5	37.0±0.4	36.9±0.5	0.210
PM	[%]	11.2±1.5	11.2±1.5	11.1±1.5	11.2±1.5	11.2±1.6	11.2±1.7	0.506
SLM	[%]	52.8±7.1	52.7±6.9	52.4±7.1	52.9±7.1	53.1±7.4	53.0±7.7	0.638
FFM	[%]	56.1±7.6	55.9±7.4	55.6±7.6	56.2±7.6	56.4±7.9	56.3±8.2	0.620
SMM	[%]	31.8±4.6	31.7±4.4	31.5±4.6	31.8±4.6	31.9±4.8	32.0±5.0	0.497
PBF	[%]	13.3±5.0	13.1±4.7	13.6±4.9	13.2±4.6	13.2±5.2	13.6±6.0	0.800
FAT	[kg]	8.6±3.4	8.5±3.4	8.8±3.5	8.5±3.1	8.5±3.5	8.8±4.0	0.831
VFA	[cm^2^]	28.0±15.3	27.3±15.7	29.1±16.2	27.7±14.1	27.1±15.3	28.9±16.9	0.353

The body composition index measurements taken before each session are presented as mean ± SD; no significant difference was observed in the body composition indices between sessions

*Rt* right, *ICW* intracellular water, *ECW* extracellular water, *TBW* total body water, *PM* protein mass, *SLM* soft lean mass, *FFM* fat-free mass, *SMM* skeletal muscle mass, *PBF* percent body fat, *VFA* visceral fat area

### Changes in skin temperature and auditory canal temperature due to hot pack use (Figs. 2, 3, and 4)

The time course of changes in the skin and auditory canal temperature with the use of hot packs is shown in Fig. 1. The auditory canal temperature showed no significant changes during any of the sessions. In the sessions where the hot pack was used on the right shoulder, the right upper arm and pronotum showed a significant increase in body temperature. However, no significant changes were observed in the other areas. In addition, the increased skin temperature of the right upper arm and pronotum decreased slowly after the hot pack was removed and returned to the pre-application value after 20 min.

**Fig. 2:**
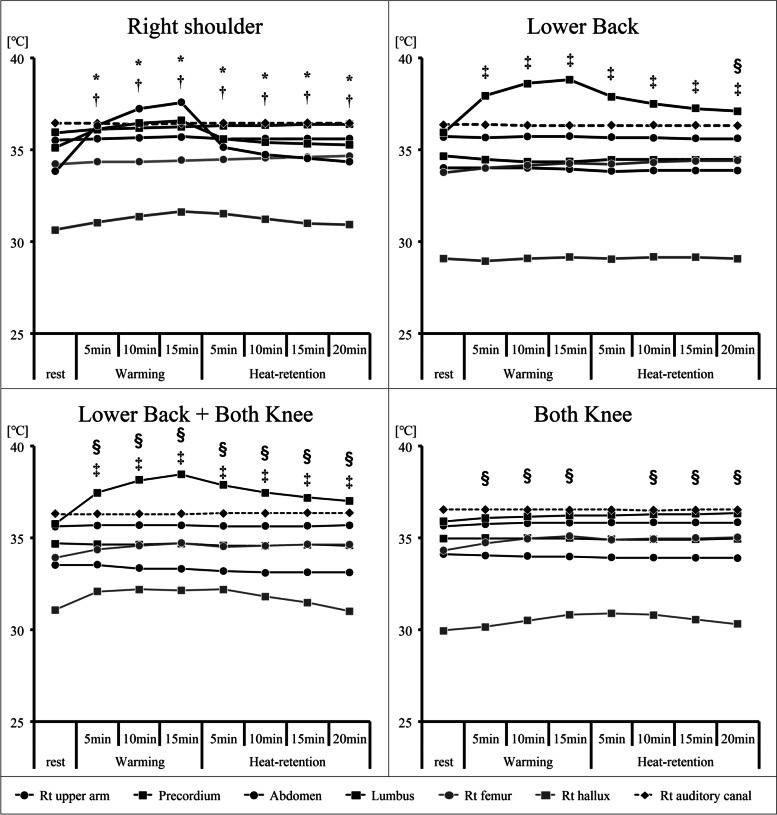
Temperature changes in each body part according to the heated area. The body temperature response during and after hot pack treatment is shown for each measurement site. All data represent averages over 10 s of elapsed time. †*p* < 0.05 vs. rest (symbols are color-coded for each measurement area). Lt, left; Rt, right

**Fig. 3:**
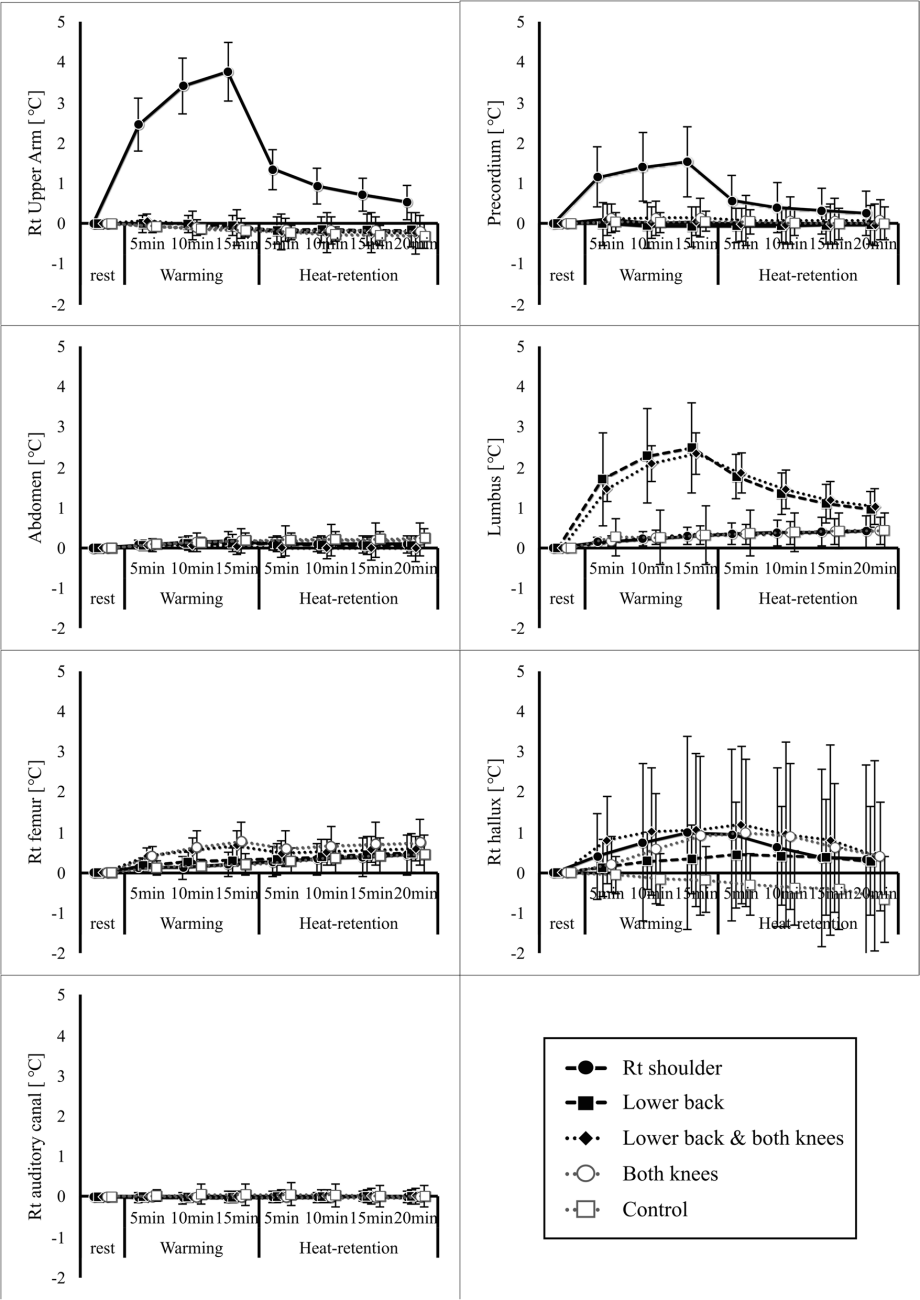
Temperature changes at each measurement site from the resting state. The body temperature response during and after hyperthermia treatment is shown for each measurement site from the resting state. All data represent averages over 10 s of elapsed time

**Fig. 4:**
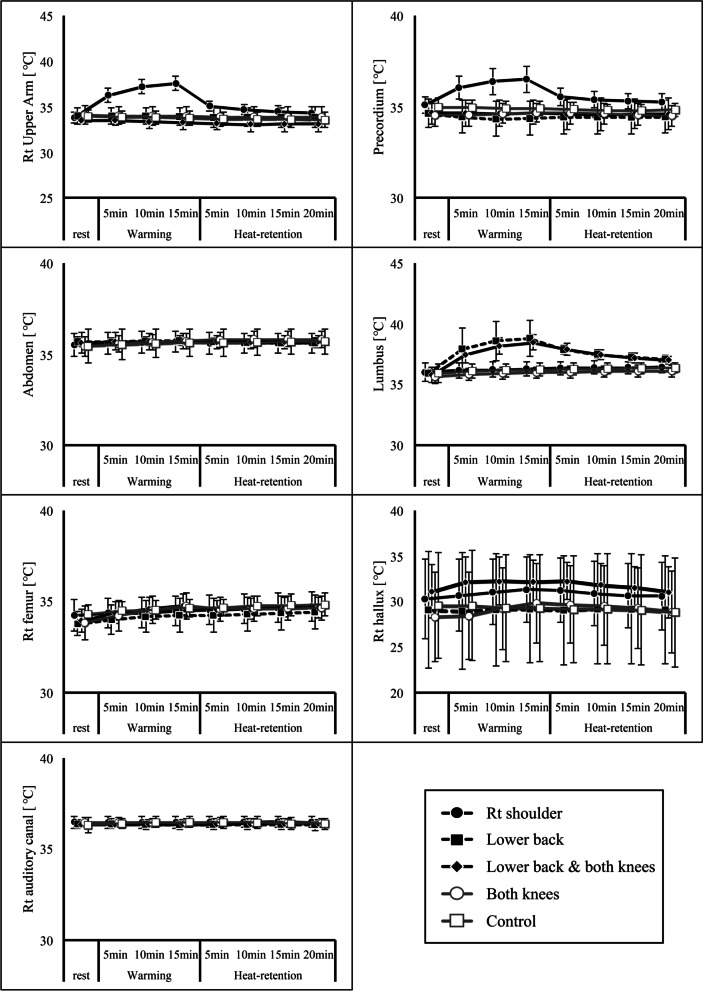
Temperature changes at each measurement site. The temperature changes at each measurement site are shown for the differences in relation to the hot pack application site. All data represent averages over 10 s of elapsed time

Graphs showing the degree of change with respect to the beginning of rest for each hot pack application method and the change in temperature for each method at each measurement site are shown in the Figs. 3 and 4.

### Relationships between body temperature change and body composition indices

The ECW and ECW/TBW (total) measured by the Inbody S10 showed a significant correlation (*r* = 0.496, *p* = 0.037 and *r* = 0.583, *p* = 0.011, respectively) with the increase in the skin temperature of the right upper arm when the hot pack was applied to the right shoulder. In the lower abdomen, temperature change and ECW/TBW (total) showed a significant correlation (*r* = 0.495, *p* = 0.037), although the average increase in temperature was not significant. In addition, during the resting period after the application of the hot pack on the right shoulder, the decrease in lumbus temperature (0.13 ± 0.17 °C) showed a significant negative correlation with the water content of both lower extremities (segmental water (RL): *r* = −0.558, *p* = 0.016; segmental water (LL): *r* = −0.547, *p* = 0.019; segmental lean (RL): *r* = −0.555, *p* = 0.017; segmental lean (LL): *r* = −0.545, *p* = 0.019; segmental ICW (RL): *r* = −0.544, *p* = 0.020; segmental ICW (LL): *r* = −0.538, *p* = 0.021; segmental ECW (RL): *r* = −0.572, *p* = 0.013; segmental ECW (LL): *r* = −0.553, *p* = 0.017). On the other hand, no association was found with indices related to body fat, such as VFA and FAT.

In the sessions where hot packs were placed on the lower back and lower back plus both knee fossae, no association was found between the temperature increases in the lumbus and femur regions and body composition indices. There was a negative correlation between temperature changes in the right hallux and body water-related indices, such as ICW (*r* = −0.551, *p* = 0.018) and TBW (*r* = −0.554, *p* = 0.017). Similar associations were found for indices related to total body water content (segmental water, segmental lean, segmental ICW, and segmental ECW) without the body site characteristics of the upper limb, lower limb, and trunk.

In the session where hot packs were placed on both knee fossae, no significant temperature increase was observed in either the lower abdomen or lumbus, but FAT, PBF, and VFA were significantly correlated with temperature changes in the lower abdomen and lumbus (for the lower abdomen, *r* = 0.636, *p* = 0.11, *r* = 0.622, *p* = 0.017, and *r* = 0.595, *p* = 0.024, respectively; for the lumbus, *r* = 0.552, *p* = 0.030, *r* = 0.525, *p* = 0.034, and *r* = 0.534, *p* = 0.029, respectively).

### Hot pack weight, temperature change, and the estimated amount of heat

The dry weight of the hot pack (after being dried for at least 1 week) and the wet weight (after soaking in warm water at 80 °C for more than 24 h) were 1243.3 ± 0.96 g and 2140.5 ± 3.5 g respectively. The temperature on the surface of the hot pack was 59.8 ± 2.7 °C at the start of heating, 58.2 ± 2.6 °C after 5 min, 56.8 ± 2.5 °C after 10 min, and 55.6 ± 2.4 °C after 15 min (*p* < 0.001). The estimated amount of heat of the hot pack, calculated from the weight, specific heat, and temperature change of the hot pack, was approximately 25,200 J (6.04 kcal).

## Discussion

Most past studies that evaluated the conduction of heat to the human body mainly described the temperature changes and heat depth at the hot pack application sites. However, few reports have attempted to determine the temperature changes of the whole body during and after superficial thermal therapy, including therapy with hot packs. Thus, the present study is one of the few studies that evaluated changes in the skin temperature of the whole body in addition to the sites where the hot pack was applied and examined the relationship of these changes with body composition indices. The results suggested that the increase and decrease in skin temperature were related to body water content, such as ECW, in the body composition index.

### The relationship between body composition indices and changes in body temperature

While evaluating the results of this study, we assumed that a temperature increase would be related to ECW and body water content because of the ease of thermal conductivity, or that the temperature decrease would be related to body fat content because of the difficulty in heat dissipation and the ease of heat retention. However, the relationship between body composition indices and the temperature changes in each body part with hot pack application differed from those in previous studies [18]. The temperature increase in the lower abdomen and back when the hot pack was placed on both knee fossae was positively correlated with PBF, FAT, and VFA. Thus, the higher the body fat, the higher the temperature.

When hot packs were placed on the lower back and both knee fossae, the temperature increase in the left hallux was negatively correlated with ICW, TBW, and other indices related to water content without relation to regional properties. A similar trend was observed when hot packs were applied to both knee fossae, although the trend was not statistically significant. Thus, the higher the body water content, the more difficult it was to increase the temperature. This may be related to the fact that the heat dissipation from the surface of the body was greater than the heat retention effect of fat because the body fat percentage of the patients in this study was low.

However, several factors may have contributed to the inconsistent findings regarding the relationship between body composition indices and temperature changes. First, most participants had a standard body shape, and the individual differences were small. Second, the amount of heat energy provided by the hot pack to the body was small; thus, there was little change.

### Changes in the skin temperature of the whole body with the use of a single hot pack

The hot pack only increased the temperature at the heated site on the very near side. When the hot pack was applied to the right shoulder, the temperature rise at the right upper arm was 3.77 ± 0.74°C, and for the lower back, the rise at the lumbus was 2.44 ± 0.86°C. The reasons for the difference in the temperature rise by site is thought to be because of the difference in temperature between the sites where the hot pack was applied and the hot pack, and differences in the mass of the warmed site.

We tried to calculate how much heat provided by the hot pack would be needed to raise the temperature of the upper extremities or lower back, based on a comparison of the estimated amount of heat provided by the hot pack with the amount of heat required to raise the temperature calculated from the average segment weight [29], specific heat of the body, and temperature change. However, for the lower back, it was difficult to compare the amount of heat with that of the hot pack because not all of the trunk increased in temperature (there was almost no increase in temperature in the lower abdomen). In addition, for the upper arm, it was difficult to calculate since this study only measured the temperature change of the skin temperature, not the entire upper arm, and the hot pack also dissipates heat from the side that is not in contact with the body.

Moreover, the body continuously absorbs and diffuses heat to and from the atmosphere, except for the site warmed by the hot pack [30, 31]. It is possible that the higher body water content was more involved in heat diffusion since the temperature increase was suppressed.

Thermal therapies such as saunas [32, 33], foot baths [34, 35], and infrared treatments [36] are effective in increasing deep-body temperature. However, to achieve an increase in deep-body temperature and transfer heat energy to the whole body, heat therapy with hot packs should be performed with the provision of heat energy to a wider area and an appropriate method of heat transfer. This can be achieved by increasing the number of hot packs used and applying them to different parts of the body, among other methods. An alternative approach to transfer heat energy from the heat packs to deeper parts of the body can also be devised (e.g., by developing an appropriate heat retention method).

### Potential for whole-body thermal therapy using hot packs

The benefits of whole-body thermal therapy are manifold, including those for patients with chronic heart failure. In these patients, a temporary increase in deep-body temperature can facilitate improved vascular function and autonomic nerve function, in addition to providing other benefits. The authors have previously reported the possibility of performing whole-body thermal therapy using hot packs and aluminum sheets to create an environment that can provide whole-body thermal therapy easily without requiring equipment such as a sauna [24]. In that study, the participants’ deep-body temperature could be increased by placing three hot packs on their back, knee fossa, and lower abdomen and covering their entire body with aluminum sheets. However, since that study only examined the use of three hot packs, it was unclear how many hot packs were necessary to increase deep-body temperature and whether this number was affected by body composition.

The results of this study clearly indicate that the changes in the skin temperature directly below the hot pack did not affect the body composition indices in participants with a standard body shape and a BMI of approximately 22 kg/m^2^. In addition, it became more difficult to increase the skin temperature distal to the application site as the amount of body water increased. Furthermore, the hot pack alone did not increase the temperature of the auditory canal or the skin temperature outside the applied area, and the use of the hot pack alone was insufficient to increase the deep-body temperature.

Therefore, we concluded that the use of a hot pack alone does not provide the heat energy necessary to increase the deep-body temperature of the human body or the body temperature at sites other than the application site. In other words, to increase the deep-body temperature using a hot pack, the heat of the hot pack should be accompanied by an environment limiting heat dissipation from the body and loss of heat from the hot pack (radiant heat). Consequently, in the future, we hope to adjust the number of hot packs and the sites where they should be used, as well as to identify more efficient ways of limiting the heat energy to the body.

### Study limitations and scope

This study is the first to investigate the temperature changes in various body parts during hot pack application. However, this study has several limitations. First, the BIA method for body composition measurement did not measure the amount or percentage of body fat in each body part, although it did measure body water and muscle mass. The other limitation was that subcutaneous fat thickness was not measured. Therefore, we were unable to examine the relationship between temperature change and body fat in body parts. Second, because we had not taken detailed body measurements of the limbs, we could not come up with a thorough estimate of the amount of heat provided by the hot packs. Third, the number of hot packs used in this study was one or two per session, which differed from the number of hot packs used in whole-body thermal therapy in our previous study. Therefore, we could not determine the number of hot packs necessary to increase the deep-body temperature.

## Conclusions

Hot pack use did not increase the auditory canal temperature, which reflects the deep-body temperature. In healthy men with a normal body size, the skin temperature increase was significant only in the vicinity of the application site; however, the skin temperature change was negatively correlated with the amount of body water content in the whole body. In other words, the higher the body water content, the more diffuse the skin temperature was.

## Supplementary Information

**Additional file 1.** Relationships between changes in body temperature and body composition indices with significant associations.

**Additional file 2.** Correlations between the body composition indices of each body segment and changes in body temperature.

## Data Availability

The datasets generated and analyzed during the current study are available in the “figshare” repository, 10.6084/m9.figshare.19112528.
