# Supplementary material for: Relationship between body composition indices and changes in body temperature due to hot pack use
Source: J Physiol Anthropol. 2022 Nov 11;41:40. doi: 10.1186/s40101-022-00313-0 (PMC9650860; doi:10.1186/s40101-022-00313-0)

Additional file 1-1. Figures for the relationships between changes in body temperature and body composition indices with significant associations.

Hot pack application site
- Right shoulder -

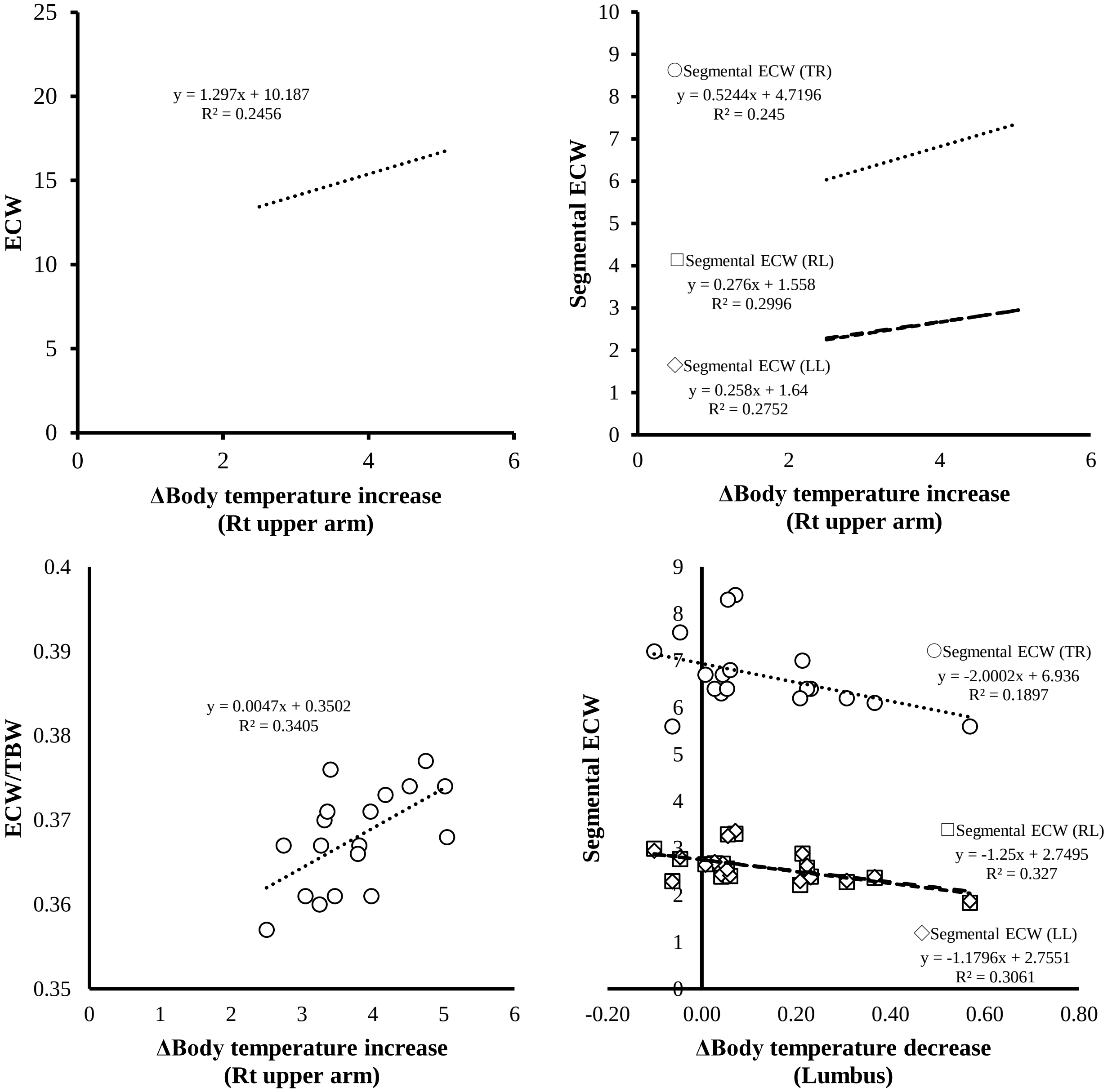

Additional file 1-2. Figures for the relationships between changes in body temperature and body composition indices with significant associations.

Hot pack application site
- Low back -

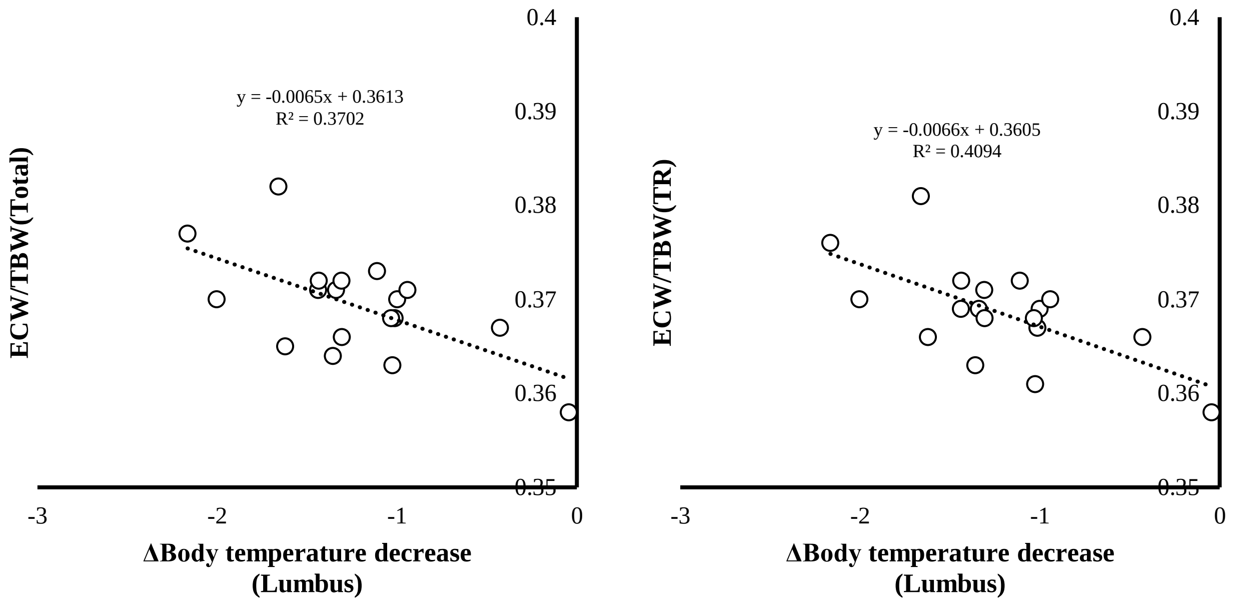

Additional file 1-3. Figures for the relationships between changes in body temperature and body composition indices with significant associations.

Hot pack application site
- Low back & Both knee -

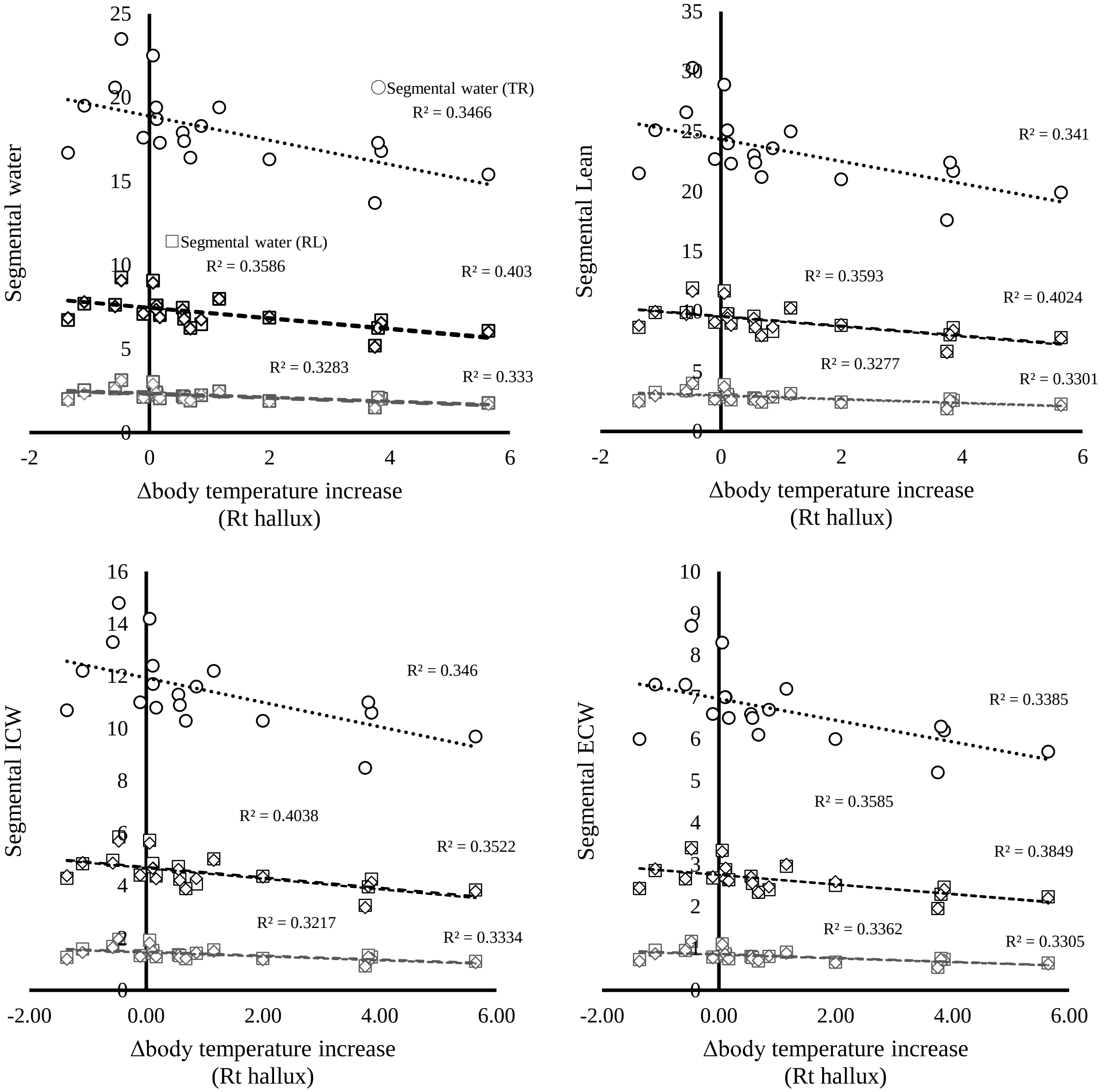

Additional file 1-4. Figures for the relationships between changes in body temperature and body composition indices with significant associations.

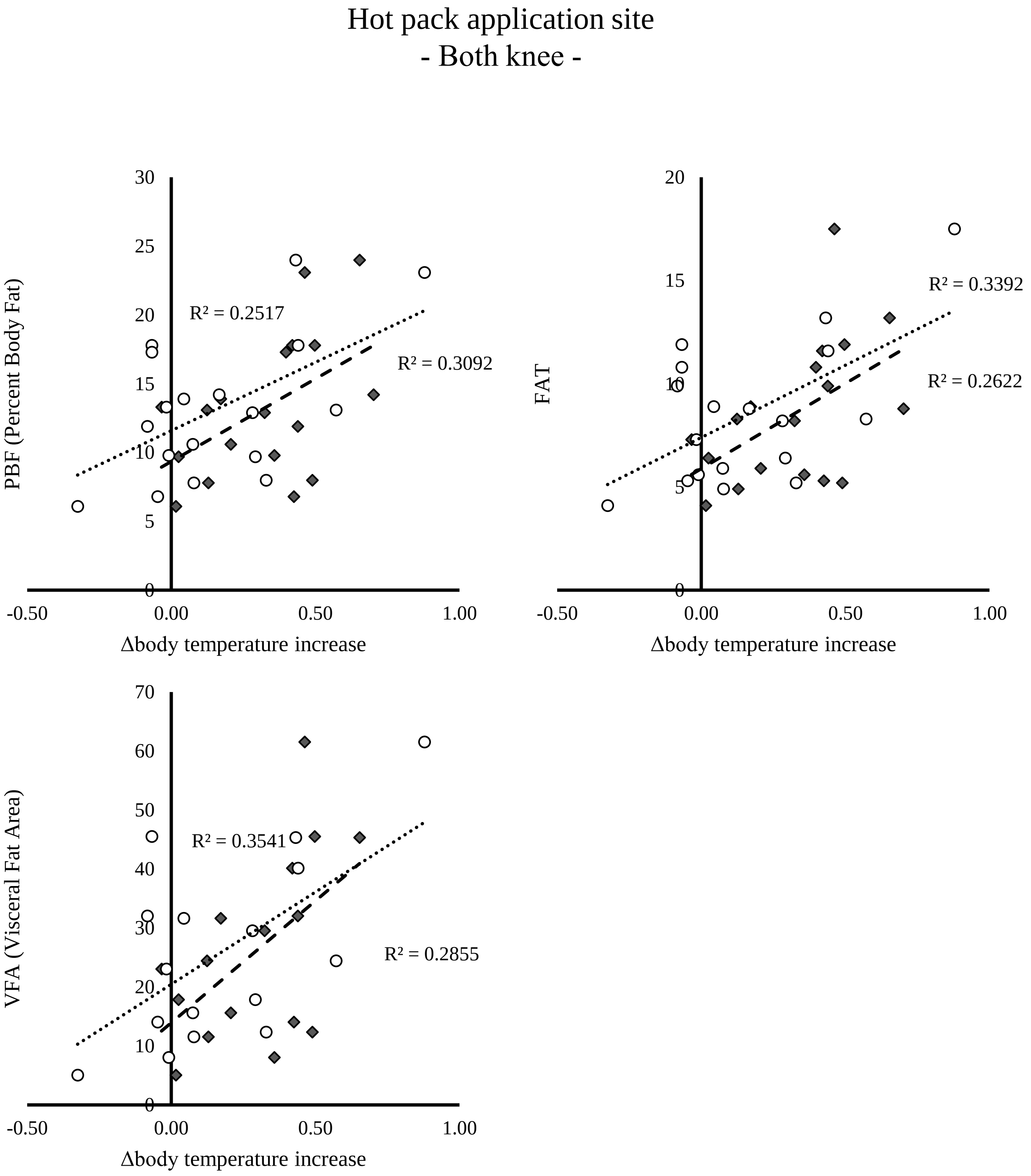

Supplement: Supplementary file 1 — Additional file 1. Relationships between changes in body temperature and body composition indices with significant associations. [file 40101_2022_313_MOESM1_ESM.pdf]
