# Supplementary material for: Relationship between body composition indices and changes in body temperature due to hot pack use
Source: J Physiol Anthropol. 2022 Nov 11;41:40. doi: 10.1186/s40101-022-00313-0 (PMC9650860; doi:10.1186/s40101-022-00313-0)
Supplement: Supplementary file 2 — Additional file 2. Correlations between the body composition indices of each body segment and changes in body temperature. [file 40101_2022_313_MOESM2_ESM.pdf]

Additional file 2-1 Correlation between body composition indices of each body segment and changes in body temperature.

Hot pack application site
- Right shoulder -

	Rt upper arm		Precordium		Abdomen		Lumbus		Rt femur		Rt hullex		Rt auditory canal	
	Warming	Heat-retention	Warming	Heat-retention	Warming	Heat-retention	Warming	Heat-retention	Warming	Heat-retention	Warming	Heat-retention	Warming	Heat-retention
WEIGHT	0.451	0.013	0.037	0.133	-0.078	-0.213	-0.220	-0.343	0.201	0.103	-0.131	0.191	0.057	-0.173
ICW	0.375	0.011	-0.094	0.254	-0.171	-0.161	-0.328	-0.452	0.221	0.063	-0.285	0.240	0.121	-0.125
ECW	0.496	-0.038	-0.038	0.200	-0.079	-0.138	-0.343	-0.469	0.187	0.077	-0.252	0.178	0.151	-0.067
TBW	0.420	-0.007	-0.074	0.236	-0.138	-0.153	-0.335	-0.460	0.210	0.068	-0.275	0.219	0.133	-0.105
PM	0.367	0.015	-0.091	0.249	-0.181	-0.178	-0.325	-0.452	0.224	0.063	-0.271	0.241	0.125	-0.133
MM	0.375	-0.016	-0.020	0.181	-0.136	-0.156	-0.229	-0.359	0.208	0.102	-0.262	0.268	0.094	-0.176
FAT	0.186	0.038	0.244	-0.193	0.128	-0.161	0.182	0.161	0.012	0.089	0.280	-0.042	-0.138	-0.156
SLM	0.408	-0.002	-0.080	0.240	-0.145	-0.156	-0.332	-0.457	0.214	0.068	-0.276	0.224	0.129	-0.113
FFM	0.407	-0.003	-0.074	0.235	-0.146	-0.157	-0.326	-0.451	0.214	0.071	-0.275	0.228	0.127	-0.116
SMM	0.377	0.007	-0.094	0.252	-0.172	-0.162	-0.327	-0.451	0.222	0.063	-0.284	0.239	0.127	-0.127
DLM	0.370	0.007	-0.073	0.234	-0.165	-0.166	-0.300	-0.426	0.222	0.079	-0.274	0.253	0.111	-0.143
PBF	0.081	0.032	0.273	-0.264	0.183	-0.084	0.297	0.324	-0.093	0.064	0.339	-0.110	-0.211	-0.099
BMI	0.251	0.151	0.259	-0.044	-0.162	-0.301	0.003	-0.083	0.169	0.202	-0.087	0.393	-0.055	-0.235
Segmental Water(RA)	0.391	0.074	-0.070	0.229	-0.179	-0.185	-0.307	-0.438	0.214	0.082	-0.284	0.247	0.111	-0.096
Segmental Water(LA)	0.453	0.021	-0.052	0.200	-0.131	-0.193	-0.287	-0.415	0.192	0.051	-0.250	0.182	0.100	-0.084
Segmental Water(TR)	0.434	0.019	-0.083	0.218	-0.142	-0.194	-0.295	-0.431	0.214	0.053	-0.243	0.175	0.108	-0.106
Segmental Water(RL)	0.471	-0.094	-0.171	0.292	-0.093	-0.083	-0.428	-0.558	0.175	-0.011	-0.226	0.060	0.190	-0.056
Segmental Water(LL)	0.449	-0.092	-0.158	0.283	-0.077	-0.098	-0.424	-0.547	0.217	0.026	-0.228	0.087	0.205	-0.070
ECW/TBW(Total)	0.583	-0.205	0.279	-0.252	0.495	0.147	-0.135	-0.115	-0.217	0.075	0.172	-0.314	0.051	0.366
ECW/TBW(TR)	0.560	-0.225	0.301	-0.310	0.461	0.093	-0.011	-0.017	-0.252	0.009	0.172	-0.334	-0.018	0.283
Segmental Lean(RA)	0.393	0.071	-0.073	0.230	-0.180	-0.188	-0.305	-0.439	0.209	0.075	-0.287	0.244	0.111	-0.100
Segmental Lean(LA)	0.448	0.021	-0.056	0.201	-0.129	-0.197	-0.277	-0.405	0.195	0.050	-0.247	0.177	0.093	-0.091
Segmental Lean(TR)	0.424	0.025	-0.086	0.224	-0.156	-0.197	-0.299	-0.435	0.212	0.047	-0.247	0.182	0.106	-0.107
Segmental Lean(RL)	0.459	-0.089	-0.178	0.298	-0.103	-0.083	-0.424	-0.555	0.179	-0.014	-0.232	0.066	0.187	-0.064
Segmental Lean(LL)	0.437	-0.087	-0.160	0.285	-0.088	-0.102	-0.422	-0.545	0.221	0.027	-0.230	0.096	0.204	-0.075
Segmental ICW(RA)	0.377	0.061	-0.074	0.223	-0.195	-0.204	-0.294	-0.428	0.217	0.069	-0.281	0.245	0.099	-0.112
Segmental ICW(LA)	0.450	0.010	-0.055	0.197	-0.139	-0.207	-0.274	-0.406	0.193	0.041	-0.255	0.181	0.104	-0.102
Segmental ICW(TR)	0.396	0.028	-0.103	0.239	-0.167	-0.198	-0.295	-0.426	0.224	0.046	-0.258	0.195	0.101	-0.124
Segmental ICW(RL)	0.422	-0.069	-0.200	0.319	-0.132	-0.089	-0.412	-0.544	0.187	-0.023	-0.254	0.087	0.177	-0.089
Segmental ICW(LL)	0.400	-0.072	-0.167	0.292	-0.120	-0.112	-0.414	-0.538	0.238	0.031	-0.239	0.124	0.203	-0.094
Segmental ECW(RA)	0.413	0.095	-0.062	0.236	-0.150	-0.152	-0.326	-0.454	0.207	0.105	-0.289	0.248	0.133	-0.068
Segmental ECW(LA)	0.457	0.040	-0.047	0.204	-0.116	-0.167	-0.308	-0.428	0.190	0.069	-0.240	0.182	0.093	-0.052
Segmental ECW(TR)	0.495	0.002	-0.046	0.180	-0.096	-0.185	-0.293	-0.435	0.195	0.063	-0.215	0.138	0.118	-0.074
Segmental ECW(RL)	0.547	-0.135	-0.119	0.241	-0.024	-0.070	-0.447	-0.572	0.152	0.009	-0.175	0.012	0.208	0.000
Segmental ECW(LL)	0.525	-0.124	-0.141	0.263	-0.004	-0.075	-0.435	-0.553	0.177	0.017	-0.206	0.024	0.204	-0.028
BCM	0.375	0.008	-0.097	0.255	-0.172	-0.160	-0.327	-0.452	0.223	0.063	-0.287	0.241	0.126	-0.128
BMC	0.379	-0.020	0.009	0.168	-0.122	-0.153	-0.236	-0.351	0.201	0.109	-0.277	0.295	0.090	-0.159
VFA	0.280	0.013	0.272	-0.224	0.219	-0.113	0.177	0.156	-0.020	0.106	0.303	-0.106	-0.084	-0.100

The relationship between temperature changes and body composition indices for each session are shown. “Warming” indicates a temperature change in 15 minutes after the hot pack is applied, and “Heat-retention” indicates a temperature change for 20 minutes after the hot pack is removed. Red squares indicate significant positive correlations, and yellow squares indicate significant negative correlations.

Rt, right; ICW, intracellular water; ECW, extracellular water; TBW, total body water; PM, protein mass; MM, mineral mass; SLM, soft lean mass; FFM, fat-free mass; SMM, skeletal muscle mass; DLM, dry lean mass; PBF, percent body fat; BCM, body cell mass; BMC, bone mineral content; VFA, visceral fat area

Additional file 2-2 Correlation between body composition indices of each body segment and changes in body temperature.

Hot pack application site
- Low back -

	Rt upper arm		Precordium		Abdomen		Lumbus		Rt femur		Rt hullex		Rt auditory canal	
	Warming	Heat-retention	Warming	Heat-retention	Warming	Heat-retention	Warming	Heat-retention	Warming	Heat-retention	Warming	Heat-retention	Warming	Heat-retention
WEIGHT	0.178	0.176	0.228	-0.209	-0.167	0.095	-0.105	-0.030	-0.342	-0.173	-0.072	0.302	0.308	0.259
ICW	-0.008	0.127	0.022	-0.057	-0.211	0.110	-0.055	0.045	-0.162	-0.121	-0.149	0.204	0.469	0.448
ECW	-0.027	0.132	0.082	-0.083	-0.206	0.151	-0.083	-0.041	-0.151	-0.154	-0.209	0.250	0.407	0.447
TBW	-0.015	0.129	0.044	-0.066	-0.210	0.126	-0.065	0.014	-0.159	-0.133	-0.172	0.221	0.448	0.449
PM	-0.017	0.122	0.012	-0.051	-0.221	0.099	-0.046	0.054	-0.167	-0.116	-0.146	0.204	0.474	0.440
MM	-0.089	0.205	0.092	0.000	-0.198	0.152	-0.053	-0.078	-0.137	-0.189	-0.136	0.317	0.401	0.443
FAT	0.466	0.127	0.445	-0.366	0.063	-0.046	-0.117	-0.099	-0.464	-0.116	0.188	0.225	-0.248	-0.360
SLM	-0.014	0.128	0.040	-0.064	-0.209	0.124	-0.063	0.022	-0.158	-0.127	-0.167	0.216	0.456	0.449
FFM	-0.021	0.133	0.043	-0.059	-0.211	0.124	-0.061	0.013	-0.159	-0.135	-0.165	0.226	0.450	0.448
SMM	-0.008	0.132	0.024	-0.060	-0.209	0.109	-0.058	0.047	-0.160	-0.119	-0.148	0.204	0.469	0.447
DLM	-0.037	0.144	0.041	-0.039	-0.213	0.121	-0.048	0.010	-0.158	-0.141	-0.147	0.237	0.454	0.446
PBF	0.425	0.079	0.432	-0.359	0.097	-0.082	-0.056	-0.164	-0.387	-0.087	0.219	0.157	-0.431	-0.508
BMI	0.323	0.240	0.389	-0.246	-0.165	0.015	-0.037	-0.138	-0.451	-0.162	0.091	0.357	0.257	0.014
Segmental Water(RA)	0.110	0.037	0.064	-0.069	-0.152	0.038	-0.031	0.037	-0.127	-0.012	-0.174	0.140	0.423	0.363
Segmental Water(LA)	0.164	0.049	0.083	-0.087	-0.168	0.073	-0.036	0.000	-0.164	-0.023	-0.206	0.142	0.459	0.356
Segmental Water(TR)	0.147	0.032	0.059	-0.088	-0.140	0.052	-0.042	0.051	-0.157	-0.014	-0.190	0.126	0.444	0.366
Segmental Water(RL)	-0.120	0.138	-0.006	-0.101	-0.242	0.202	-0.119	0.073	-0.138	-0.155	-0.188	0.182	0.459	0.494
Segmental Water(LL)	-0.100	0.133	-0.006	-0.082	-0.225	0.206	-0.112	0.087	-0.143	-0.155	-0.166	0.189	0.466	0.501
ECW/TBW(Total)	-0.084	-0.028	0.381	-0.116	-0.058	0.316	-0.172	-0.608	0.069	-0.251	-0.373	0.265	-0.479	-0.050
ECW/TBW(TR)	-0.042	-0.061	0.415	-0.094	-0.047	0.299	-0.122	-0.640	0.078	-0.200	-0.408	0.241	-0.450	-0.064
Segmental Lean(RA)	0.115	0.035	0.061	-0.069	-0.150	0.035	-0.028	0.041	-0.130	-0.011	-0.173	0.136	0.424	0.361
Segmental Lean(LA)	0.170	0.048	0.076	-0.086	-0.170	0.065	-0.033	0.006	-0.167	-0.021	-0.201	0.136	0.462	0.354
Segmental Lean(TR)	0.152	0.033	0.058	-0.093	-0.136	0.044	-0.040	0.062	-0.165	-0.013	-0.184	0.121	0.453	0.363
Segmental Lean(RL)	-0.119	0.140	-0.010	-0.100	-0.242	0.197	-0.117	0.080	-0.139	-0.152	-0.182	0.179	0.464	0.493
Segmental Lean(LL)	-0.098	0.134	-0.013	-0.079	-0.224	0.200	-0.110	0.096	-0.142	-0.152	-0.160	0.186	0.470	0.502
Segmental ICW(RA)	0.133	0.020	0.054	-0.075	-0.144	0.017	-0.018	0.050	-0.119	0.016	-0.155	0.107	0.436	0.346
Segmental ICW(LA)	0.174	0.041	0.073	-0.084	-0.165	0.064	-0.027	0.016	-0.161	-0.007	-0.194	0.125	0.466	0.348
Segmental ICW(TR)	0.151	0.051	0.035	-0.080	-0.137	0.038	-0.036	0.088	-0.166	-0.010	-0.159	0.123	0.468	0.369
Segmental ICW(RL)	-0.116	0.145	-0.018	-0.097	-0.244	0.188	-0.110	0.100	-0.144	-0.144	-0.164	0.171	0.480	0.489
Segmental ICW(LL)	-0.091	0.135	-0.035	-0.065	-0.218	0.176	-0.099	0.124	-0.146	-0.141	-0.141	0.174	0.488	0.503
Segmental ECW(RA)	0.071	0.066	0.081	-0.059	-0.166	0.074	-0.052	0.012	-0.141	-0.061	-0.206	0.196	0.396	0.392
Segmental ECW(LA)	0.144	0.064	0.102	-0.092	-0.172	0.087	-0.051	-0.028	-0.169	-0.049	-0.226	0.173	0.446	0.369
Segmental ECW(TR)	0.140	0.000	0.100	-0.100	-0.143	0.076	-0.050	-0.010	-0.142	-0.021	-0.242	0.130	0.401	0.360
Segmental ECW(RL)	-0.127	0.123	0.015	-0.108	-0.235	0.227	-0.134	0.022	-0.126	-0.173	-0.229	0.200	0.416	0.498
Segmental ECW(LL)	-0.114	0.128	0.042	-0.109	-0.235	0.254	-0.133	0.026	-0.136	-0.177	-0.205	0.213	0.426	0.494
BCM	-0.008	0.131	0.024	-0.059	-0.208	0.109	-0.055	0.049	-0.164	-0.120	-0.147	0.204	0.471	0.446
BMC	-0.115	0.189	0.086	-0.005	-0.227	0.143	-0.048	-0.106	-0.150	-0.235	-0.154	0.329	0.365	0.449
VFA	0.406	0.087	0.455	-0.299	0.054	0.006	-0.126	-0.235	-0.395	-0.172	0.084	0.267	-0.346	-0.332

The relationship between temperature changes and body composition indices for each session are shown. “Warming” indicates a temperature change in 15 minutes after the hot pack is applied, and “Heat-retention” indicates a temperature change for 20 minutes after the hot pack is removed. Red squares indicate significant positive correlations, and yellow squares indicate significant negative correlations.

Rt, right; ICW, intracellular water; ECW, extracellular water; TBW, total body water; PM, protein mass; MM, mineral mass; SLM, soft lean mass; FFM, fat-free mass; SMM, skeletal muscle mass; DLM, dry lean mass; PBF, percent body fat; BCM, body cell mass; BMC, bone mineral content; VFA, visceral fat area

Additional file 2-3 Correlation between body composition indices of each body segment and changes in body temperature.

Hot pack application site
- Low back & Both knee -

	Rt upper arm		Precordium		Abdomen		Lumbus		Rt femur		Rt hullex		Rt auditory canal	
	Warming	Heat-retention	Warming	Heat-retention	Warming	Heat-retention	Warming	Heat-retention	Warming	Heat-retention	Warming	Heat-retention	Warming	Heat-retention
WEIGHT	0.188	0.126	0.086	-0.175	-0.059	-0.549	-0.016	0.199	-0.485	0.067	-0.002	-0.227	0.153	-0.213
ICW	0.252	0.288	0.032	-0.194	-0.181	-0.492	-0.162	0.163	-0.551	0.084	-0.176	-0.175	0.176	-0.113
ECW	0.228	0.253	0.086	-0.172	-0.133	-0.521	-0.118	0.097	-0.551	0.024	-0.176	-0.223	0.127	-0.110
TBW	0.244	0.276	0.052	-0.187	-0.164	-0.505	-0.147	0.140	-0.554	0.062	-0.176	-0.193	0.159	-0.112
PM	0.253	0.278	0.044	-0.196	-0.182	-0.495	-0.160	0.157	-0.551	0.084	-0.179	-0.171	0.172	-0.116
MM	0.178	0.297	0.064	-0.154	-0.078	-0.522	-0.146	0.174	-0.411	-0.022	-0.077	-0.302	0.119	-0.104
FAT	-0.120	-0.369	0.092	0.015	0.245	-0.143	0.327	0.137	0.111	0.020	0.408	-0.082	-0.007	-0.261
SLM	0.245	0.278	0.048	-0.189	-0.169	-0.502	-0.149	0.142	-0.550	0.063	-0.175	-0.191	0.160	-0.112
FFM	0.242	0.280	0.050	-0.186	-0.161	-0.505	-0.150	0.148	-0.543	0.060	-0.168	-0.199	0.160	-0.112
SMM	0.252	0.284	0.036	-0.197	-0.180	-0.492	-0.162	0.162	-0.551	0.082	-0.177	-0.177	0.171	-0.114
DLM	0.236	0.291	0.047	-0.185	-0.152	-0.504	-0.159	0.168	-0.513	0.053	-0.147	-0.213	0.161	-0.110
PBF	-0.265	-0.413	0.080	0.077	0.303	0.000	0.344	0.059	0.314	-0.022	0.404	-0.060	-0.089	-0.225
BMI	-0.016	0.088	0.070	-0.095	0.078	-0.498	0.010	0.338	-0.192	0.188	0.201	-0.243	0.252	-0.297
Segmental Water(RA)	0.232	0.235	-0.001	-0.220	-0.209	-0.488	-0.163	0.095	-0.573	0.102	-0.228	-0.144	0.195	-0.168
Segmental Water(LA)	0.199	0.251	0.034	-0.205	-0.194	-0.513	-0.129	0.106	-0.577	0.126	-0.195	-0.181	0.203	-0.166
Segmental Water(TR)	0.239	0.231	0.015	-0.216	-0.209	-0.494	-0.136	0.101	-0.589	0.105	-0.206	-0.150	0.184	-0.154
Segmental Water(RL)	0.310	0.221	0.080	-0.176	-0.151	-0.472	-0.104	0.098	-0.599	-0.006	-0.184	-0.155	0.080	-0.029
Segmental Water(LL)	0.318	0.244	0.046	-0.159	-0.152	-0.453	-0.098	0.054	-0.635	-0.005	-0.199	-0.153	0.127	0.011
ECW/TBW(Total)	-0.235	-0.256	0.247	0.141	0.313	-0.112	0.323	-0.372	0.065	-0.304	0.059	-0.248	-0.258	-0.007
ECW/TBW(TR)	-0.268	-0.225	0.232	0.165	0.349	-0.139	0.294	-0.348	0.059	-0.283	0.030	-0.296	-0.228	-0.032
Segmental Lean(RA)	0.235	0.235	-0.001	-0.219	-0.210	-0.487	-0.165	0.100	-0.572	0.108	-0.228	-0.139	0.197	-0.169
Segmental Lean(LA)	0.198	0.254	0.033	-0.210	-0.199	-0.516	-0.132	0.111	-0.575	0.130	-0.195	-0.180	0.198	-0.169
Segmental Lean(TR)	0.243	0.235	0.015	-0.215	-0.214	-0.490	-0.139	0.109	-0.584	0.114	-0.205	-0.145	0.188	-0.160
Segmental Lean(RL)	0.311	0.223	0.077	-0.176	-0.154	-0.469	-0.107	0.104	-0.599	0.000	-0.185	-0.151	0.084	-0.029
Segmental Lean(LL)	0.322	0.248	0.042	-0.163	-0.158	-0.451	-0.105	0.062	-0.634	0.001	-0.200	-0.148	0.131	0.010
Segmental ICW(RA)	0.241	0.236	-0.004	-0.213	-0.206	-0.489	-0.166	0.113	-0.567	0.117	-0.211	-0.148	0.196	-0.165
Segmental ICW(LA)	0.199	0.255	0.037	-0.205	-0.202	-0.512	-0.125	0.112	-0.577	0.141	-0.195	-0.172	0.208	-0.165
Segmental ICW(TR)	0.250	0.237	-0.006	-0.220	-0.221	-0.479	-0.148	0.121	-0.588	0.127	-0.198	-0.141	0.199	-0.155
Segmental ICW(RL)	0.308	0.236	0.058	-0.188	-0.172	-0.459	-0.124	0.126	-0.593	0.015	-0.193	-0.133	0.088	-0.034
Segmental ICW(LL)	0.329	0.263	0.023	-0.174	-0.179	-0.443	-0.132	0.083	-0.635	0.024	-0.208	-0.132	0.154	0.004
Segmental ECW(RA)	0.216	0.232	0.004	-0.231	-0.213	-0.485	-0.158	0.063	-0.580	0.075	-0.255	-0.136	0.192	-0.171
Segmental ECW(LA)	0.197	0.244	0.028	-0.206	-0.181	-0.515	-0.135	0.097	-0.575	0.102	-0.194	-0.196	0.192	-0.167
Segmental ECW(TR)	0.216	0.218	0.052	-0.207	-0.185	-0.514	-0.111	0.063	-0.582	0.064	-0.217	-0.165	0.156	-0.151
Segmental ECW(RL)	0.310	0.191	0.117	-0.153	-0.113	-0.488	-0.067	0.047	-0.599	-0.043	-0.164	-0.192	0.064	-0.021
Segmental ECW(LL)	0.292	0.206	0.083	-0.132	-0.102	-0.459	-0.037	0.004	-0.620	-0.053	-0.179	-0.186	0.077	0.023
BCM	0.255	0.286	0.034	-0.193	-0.182	-0.489	-0.163	0.163	-0.552	0.083	-0.178	-0.172	0.176	-0.111
BMC	0.184	0.303	0.080	-0.140	-0.063	-0.511	-0.160	0.207	-0.404	-0.004	-0.078	-0.302	0.120	-0.120
VFA	-0.133	-0.386	0.138	0.019	0.265	-0.180	0.363	0.032	0.072	-0.044	0.377	-0.157	-0.051	-0.268

The relationship between temperature changes and body composition indices for each session are shown. “Warming” indicates a temperature change in 15 minutes after the hot pack is applied, and “Heat-retention” indicates a temperature change for 20 minutes after the hot pack is removed. Red squares indicate significant positive correlations, and yellow squares indicate significant negative correlations.

Rt, right; ICW, intracellular water; ECW, extracellular water; TBW, total body water; PM, protein mass; MM, mineral mass; SLM, soft lean mass; FFM, fat-free mass; SMM, skeletal muscle mass; DLM, dry lean mass; PBF, percent body fat; BCM, body cell mass; BMC, bone mineral content; VFA, visceral fat area

Additional file 2-4 Correlation between body composition indices of each body segment and changes in body temperature.

Hot pack application site
- Both knee -

	Rt upper arm		Precordium		Abdomen		Lumbus		Rt femur		Rt hullex		Rt auditory canal	
	Warming	Heat-retention	Warming	Heat-retention	Warming	Heat-retention	Warming	Heat-retention	Warming	Heat-retention	Warming	Heat-retention	Warming	Heat-retention
WEIGHT	-0.273	0.198	-0.135	-0.152	-0.002	-0.157	0.167	-0.199	-0.423	0.386	0.053	0.554	0.173	-0.116
ICW	-0.253	0.310	0.039	-0.104	-0.254	-0.255	-0.080	-0.180	-0.389	0.344	0.045	0.385	0.157	-0.150
ECW	-0.247	0.365	0.036	-0.072	-0.279	-0.266	-0.051	-0.126	-0.388	0.358	0.037	0.346	0.157	-0.110
TBW	-0.252	0.331	0.038	-0.093	-0.264	-0.260	-0.069	-0.161	-0.390	0.350	0.042	0.372	0.157	-0.136
PM	-0.246	0.309	0.036	-0.103	-0.249	-0.257	-0.071	-0.178	-0.374	0.333	0.047	0.388	0.156	-0.154
MM	-0.227	0.287	-0.025	0.002	-0.209	-0.339	0.067	-0.123	-0.416	0.402	-0.038	0.375	0.090	-0.218
FAT	-0.044	-0.297	-0.375	-0.141	0.582	0.258	0.512	-0.075	-0.054	0.058	0.036	0.380	0.038	0.076
SLM	-0.251	0.325	0.037	-0.096	-0.260	-0.259	-0.071	-0.167	-0.390	0.349	0.043	0.377	0.157	-0.141
FFM	-0.249	0.325	0.032	-0.087	-0.258	-0.267	-0.060	-0.162	-0.391	0.353	0.036	0.377	0.153	-0.146
SMM	-0.248	0.311	0.036	-0.100	-0.252	-0.257	-0.077	-0.181	-0.389	0.346	0.047	0.385	0.157	-0.151
DLM	-0.241	0.309	0.017	-0.071	-0.242	-0.283	-0.037	-0.166	-0.393	0.360	0.021	0.389	0.142	-0.172
PBF	0.017	-0.357	-0.371	-0.059	0.556	0.302	0.502	0.000	0.050	-0.021	-0.060	0.206	-0.027	0.095
BMI	-0.139	0.011	-0.356	0.086	0.329	-0.092	0.354	-0.265	-0.413	0.511	-0.081	0.567	0.116	-0.174
Segmental Water(RA)	-0.224	0.304	0.089	-0.103	-0.257	-0.162	-0.165	-0.226	-0.339	0.329	0.098	0.360	0.158	-0.106
Segmental Water(LA)	-0.249	0.304	0.089	-0.132	-0.259	-0.166	-0.145	-0.210	-0.382	0.359	0.080	0.364	0.188	-0.091
Segmental Water(TR)	-0.251	0.295	0.099	-0.158	-0.264	-0.151	-0.157	-0.212	-0.354	0.317	0.118	0.371	0.180	-0.082
Segmental Water(RL)	-0.260	0.289	0.109	-0.183	-0.287	-0.317	-0.037	-0.066	-0.383	0.261	0.054	0.290	0.155	-0.137
Segmental Water(LL)	-0.273	0.315	0.129	-0.229	-0.281	-0.262	-0.076	-0.066	-0.349	0.222	0.059	0.299	0.178	-0.099
ECW/TBW(Total)	0.104	0.277	-0.063	0.186	-0.102	-0.043	0.194	0.318	0.060	0.047	-0.231	-0.298	0.041	0.216
ECW/TBW(TR)	0.116	0.187	0.025	0.209	-0.093	-0.173	0.288	0.415	0.010	0.090	-0.261	-0.388	0.010	0.134
Segmental Lean(RA)	-0.224	0.300	0.092	-0.105	-0.259	-0.164	-0.168	-0.229	-0.340	0.328	0.098	0.358	0.155	-0.112
Segmental Lean(LA)	-0.250	0.301	0.090	-0.137	-0.258	-0.159	-0.149	-0.215	-0.380	0.355	0.081	0.366	0.188	-0.090
Segmental Lean(TR)	-0.248	0.291	0.101	-0.163	-0.261	-0.146	-0.162	-0.220	-0.348	0.308	0.125	0.378	0.176	-0.086
Segmental Lean(RL)	-0.262	0.284	0.109	-0.185	-0.286	-0.320	-0.037	-0.068	-0.386	0.262	0.053	0.292	0.155	-0.142
Segmental Lean(LL)	-0.272	0.312	0.131	-0.230	-0.279	-0.263	-0.079	-0.071	-0.350	0.223	0.061	0.301	0.177	-0.104
Segmental ICW(RA)	-0.225	0.293	0.087	-0.107	-0.260	-0.155	-0.177	-0.243	-0.342	0.326	0.105	0.364	0.150	-0.114
Segmental ICW(LA)	-0.250	0.290	0.096	-0.141	-0.255	-0.156	-0.152	-0.219	-0.388	0.357	0.090	0.371	0.191	-0.092
Segmental ICW(TR)	-0.256	0.277	0.096	-0.168	-0.250	-0.128	-0.165	-0.227	-0.350	0.310	0.126	0.386	0.186	-0.077
Segmental ICW(RL)	-0.262	0.262	0.112	-0.189	-0.276	-0.328	-0.033	-0.074	-0.387	0.259	0.054	0.305	0.152	-0.162
Segmental ICW(LL)	-0.267	0.299	0.135	-0.230	-0.271	-0.266	-0.087	-0.088	-0.352	0.226	0.069	0.309	0.174	-0.118
Segmental ECW(RA)	-0.221	0.321	0.094	-0.095	-0.251	-0.173	-0.144	-0.197	-0.333	0.333	0.086	0.352	0.172	-0.092
Segmental ECW(LA)	-0.247	0.326	0.077	-0.116	-0.264	-0.182	-0.133	-0.194	-0.369	0.361	0.063	0.351	0.182	-0.088
Segmental ECW(TR)	-0.241	0.324	0.106	-0.137	-0.288	-0.190	-0.143	-0.185	-0.358	0.327	0.103	0.340	0.167	-0.092
Segmental ECW(RL)	-0.254	0.336	0.102	-0.170	-0.302	-0.292	-0.044	-0.052	-0.369	0.261	0.054	0.259	0.160	-0.089
Segmental ECW(LL)	-0.281	0.339	0.119	-0.224	-0.296	-0.252	-0.056	-0.027	-0.342	0.213	0.042	0.279	0.183	-0.066
BCM	-0.251	0.310	0.039	-0.101	-0.256	-0.257	-0.080	-0.179	-0.390	0.346	0.047	0.383	0.157	-0.150
BMC	-0.227	0.309	-0.053	0.022	-0.215	-0.354	0.085	-0.100	-0.398	0.393	-0.048	0.381	0.087	-0.213
VFA	-0.040	-0.195	-0.390	-0.081	0.528	0.260	0.513	-0.018	-0.035	0.082	0.045	0.353	0.062	0.162

The relationship between temperature changes and body composition indices for each session are shown. “Warming” indicates a temperature change in 15 minutes after the hot pack is applied, and “Heat-retention” indicates a temperature change for 20 minutes after the hot pack is removed. Red squares indicate significant positive correlations, and yellow squares indicate significant negative correlations.

Rt, right; ICW, intracellular water; ECW, extracellular water; TBW, total body water; PM, protein mass; MM, mineral mass; SLM, soft lean mass; FFM, fat-free mass; SMM, skeletal muscle mass; DLM, dry lean mass; PBF, percent body fat; BCM, body cell mass; BMC, bone mineral content; VFA, visceral fat area
